# High resolution X-ray and NMR structural study of human T-cell immunoglobulin and mucin domain containing protein-3

**DOI:** 10.1038/s41598-018-35754-0

**Published:** 2018-11-30

**Authors:** Amit K. Gandhi, Walter M. Kim, Zhen-Yu J. Sun, Yu-Hwa Huang, Daniel A. Bonsor, Eric J. Sundberg, Yasuyuki Kondo, Gerhard Wagner, Vijay K. Kuchroo, Gregory Petsko, Richard S. Blumberg

**Affiliations:** 1 Division of Gastroenterology, Department of Medicine, Brigham and Women’s Hospital, Harvard Medical School, 75 Francis Street, Boston, MA 02115 USA; 2 Department of Biological Chemistry and Molecular Pharmacology, Harvard Medical School, 240 Longwood Avenue, Boston, MA 02115 USA; 3 Institute of Human Virology, School of Medicine, University of Maryland, 725 W Lombard St, Baltimore, MD 21201 USA; 4 Evergrande Center for Immunologic Diseases and Ann Romney Center for Neurologic Diseases, Harvard Medical School and Brigham and Women’s Hospital, 77 Avenue Louis Pasteur, Boston, MA 02115 USA; 5 Department of Neurology and Feil Family Brain and Mind Research Institute, Weill Cornell Medical College, New York, NY 10021 USA; 6 Present Address: Department of Cancer Biology, Dana-Farber Cancer Institute, Boston, MA 02215 USA; 7 Department of Medicine, School of Medicine, University of Maryland, Baltimore, MD 21201 USA; 8 Department of Microbiology and Immunology, School of Medicine, University of Maryland, Baltimore, MD 21201 USA; 9 Present Address: Division of Gastroenterology, Department of Internal Medicine, Graduate School of Medicine, Kobe University, Kobe, 650-0017 Japan

## Abstract

T-cell immunoglobulin and mucin domain containing protein-3 (TIM-3) is an important immune regulator. Here, we describe a novel high resolution (1.7 Å) crystal structure of the human (h)TIM-3 N-terminal variable immunoglobulin (IgV) domain with bound calcium (Ca^++^) that was confirmed by nuclear magnetic resonance (NMR) spectroscopy. Significant conformational differences were observed in the B-C, C′-C″ and C′-D loops of hTIM-3 compared to mouse (m)TIM-3, hTIM-1 and hTIM-4. Further, the conformation of the C-C′ loop of hTIM-3 was notably different from hTIM-4. Consistent with the known metal ion-dependent binding of phosphatidylserine (PtdSer) to mTIM-3 and mTIM-4, the NMR spectral analysis and crystal structure of Ca^++^-bound hTIM-3 revealed that residues in the hTIM-3 F-G loop coordinate binding to Ca^++^. In addition, we established a novel biochemical assay to define hTIM-3 functionality as determined by binding to human carcinoembryonic antigen cell adhesion molecule 1 (CEACAM1). These studies provide new insights useful for understanding and targeting hTIM-3.

## Introduction

T-cell immunoglobulin and mucin domain containing protein-3 (TIM-3), also known as Hepatitis A virus cellular receptor 2 (HAVCR2), is involved in regulating innate and adaptive immunity and has thus emerged as a target for numerous therapeutics that are under clinical development. There are three TIM family members in humans (TIM-1, TIM-3, and TIM-4) and eight members in mice (TIM-1 to TIM-8)^1–11^. TIM-3 is expressed on various cell types such as T-helper 1 (Th1) lymphocytes, dendritic cells and natural killer (NK) cells^3,8,12,13^. A major feature of TIM-3 is its potential role as a checkpoint inhibitor in immune responses to tumors, as well as its involvement in chronic viral infections^3,7,14–16^. Human (h)TIM-3 consists of a membrane distal variable immunoglobulin (IgV)-like domain involved in ligand interactions, a membrane proximal mucin domain and a transmembrane domain that is connected to an intracellular cytoplasmic tail involved in phosphotyrosine-dependent signaling^2,9,17,18^. Previous structural and biochemical studies have shown that mouse (m)TIM-3^2^ and mTIM-4^19^ bind phosphatidylserine (PtdSer) at the GFCC′ face of the IgV domain in a calcium (Ca^++^) dependent manner. PtdSer has also been implicated as a ligand for hTIM-3^10^ although a crystal structure depicting this association has yet to be solved. The IgV-like domain of hTIM-3 has also been demonstrated to bind other ligands such as carcinoembryonic antigen cell adhesion molecule 1 (CEACAM1)^4^, high mobility group protein B1 (HMGB1)^20^ and galectin-9^1,9,18^. The study of these interactions suggests that hTIM-3 may exhibit overlapping but distinct structural characteristics that determine specificity for binding of these unique ligands that all coalesce on the IgV-domain. However, the specific conformational details of hTIM-3 have been too elusive to date to allow for a comprehensive understanding of these ligand interactions. Here we report the high resolution co-crystal structure of hTIM-3 IgV-domain with bound Ca^++^ that provides a structural basis for understanding hTIM-3 at an atomic level.

## Results

### Overall structure and NMR validation of hTIM-3

**Figure 1:**
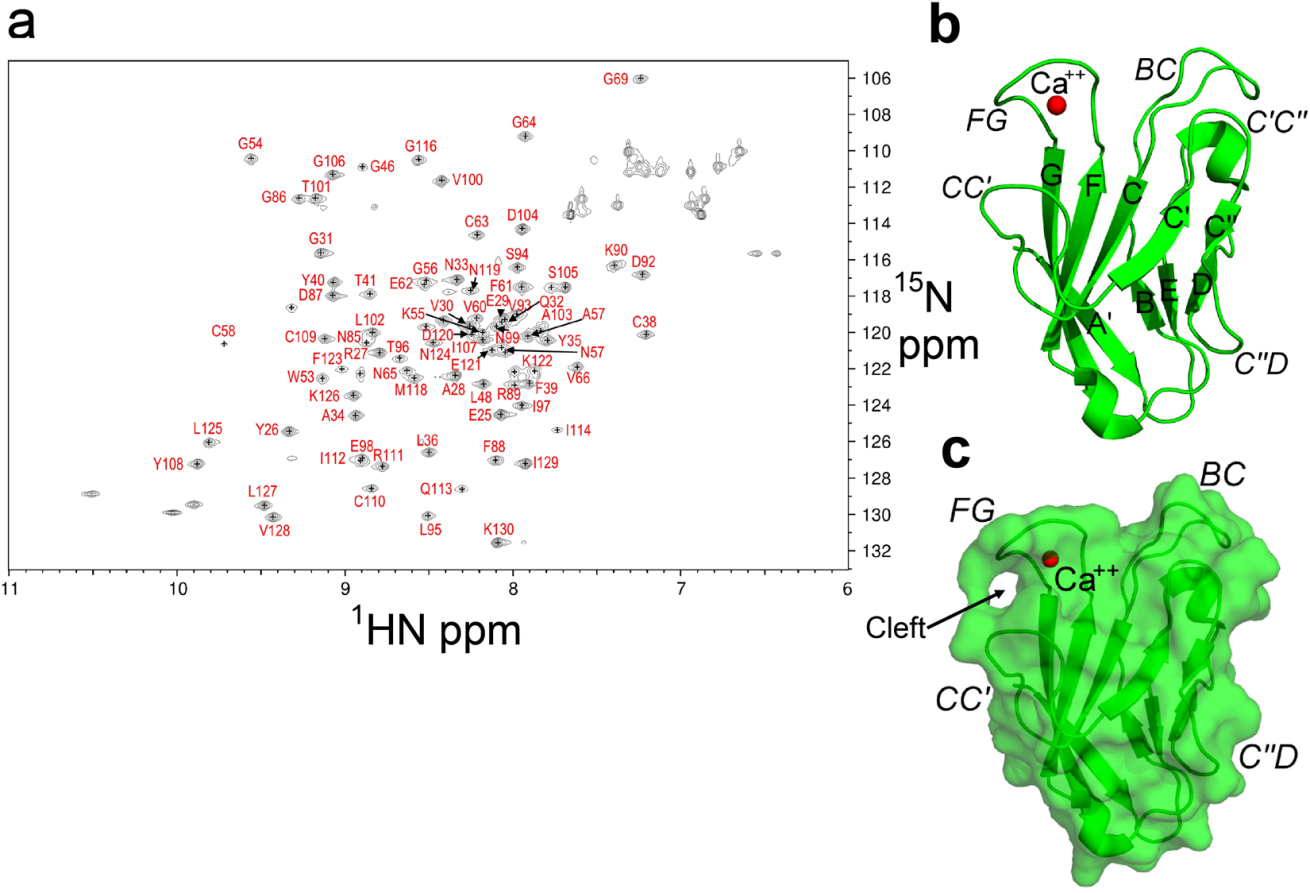
Crystal structure and NMR validation of the IgV domain of hTIM-3. (**a**) ^15^N-HSQC of ^15^N/^13^C labeled hTIM-3 acquired at 25 °C on an Agilent 700-MHz spectrometer, with amino acids assignments shown in red. (**b**) Ribbon diagram of the hTIM-3 IgV domain crystal structure with bound Ca^++^ in red. The β strands are labeled with uppercase letters and loops are highlighted in italics. (**c**) Surface view of Ca^++^ bound hTIM-3. The β strands and loops are labeled as above.

We first characterized the proper folding of purified recombinant hTIM-3 protein in its calcium bound state. Previous studies have demonstrated a melting temperature (T_m_) for unbound hTIM-3 of approximately 45 °C^10^, which we confirmed by differential scanning fluorimetry. Titration of calcium increased the melting temperature consistent with increased stability of the Ca^++^-bound hTIM-3 complex (Supplementary Fig. 1a,b). In addition, we observed an excellent global fold structure of our purified Ca^++^-bound hTIM-3 in solution as judged by the large spread of assigned amide resonance peaks in the ^15^N-heteronuclear single quantum coherence (HSQC) spectrum of ^15^N/^13^C-labeled hTIM-3 protein, which consisted mainly of extended beta-strands (Fig. 1a). To obtain the structural details of hTIM-3 at an atomic level, we therefore determined the crystal structure of the Ca^++^-bound hTIM-3 IgV domain at 1.7 Å resolution (PDB code 6DHB, Table 1). This structure exhibited features common to the TIM family members^2,9–11,21^ by possessing a two anti-parallel β-sheet sandwich formed from front AGFCC′C″ and back BED faces, respectively, which were linked by the B-C, E-F, C″-D and A-B loops (Fig. 1b,c).

**Table 1:** Crystal information, data collection and refinement parameters.

	hTIM-3 with bound Ca^++^
**Data collection statistics**	
Space Group	P 212121
Cell constants	42.31 Å, 43.06 Å, 51.72 Å
a, b, c, α, β, γ	90°, 90°, 90°
Resolution (Å)*	33–1.70 (1.73–1.70)
No. of measurements*	98221 (2181)
Unique reflections*	10823(526)
I/sigma I*	31.1 (10.5)
Completeness (%)*	99.2 (90.5)
Redundancy*	9.1 (4.1)
*R*_*merge*_ (%) ^a,^*	4.6 (8.5)
*CC*_1/2_*	0.999 (0.989)
**Structure refinement**	
*R*_*work*_ (%)^b,^*	15
*R*_*free*_ (%)^c,^*	18.1
**No. atoms**	
Protein/Water	856/100
**R.m.s. deviations**	
Bond-lengths (Å)	0.023
Bond-angles (°)	2.055
**Ramachandran plot**	
Most favored regions (%)	94
Additional allowed regions (%)	6
Disallowed (%)	0
**B-factors (Å** ^**2**^ **)**	
Protein	12.6
Water	22.8
Ca^++^	10.7

^a^*R*_*merge*_ = ∑|*I*−〈*I*〉|/∑(*I*), where *I* is the observed intensity and 〈*I*〉 is the weighted mean of the reflection intensity.

^b^*R*_*work*_ = ∑||*F*_o_|−*F*_c_||/∑|*F*_o_|, where *F*_o_ and *F*_c_ are the observed and calculated structure

factor amplitudes, respectively.

^c^R_*free*_ is the crystallographic *R*_*work*_ calculated with 5% of the data that were excluded

from the structure refinement.

^*^Values in the parentheses are for highest resolution shell.

### Structural comparison with mTIM-3 and conformational flexibility of hTIM-3 B-C loop, C″-D loop and GF-CC′ cleft

**Figure 2:**
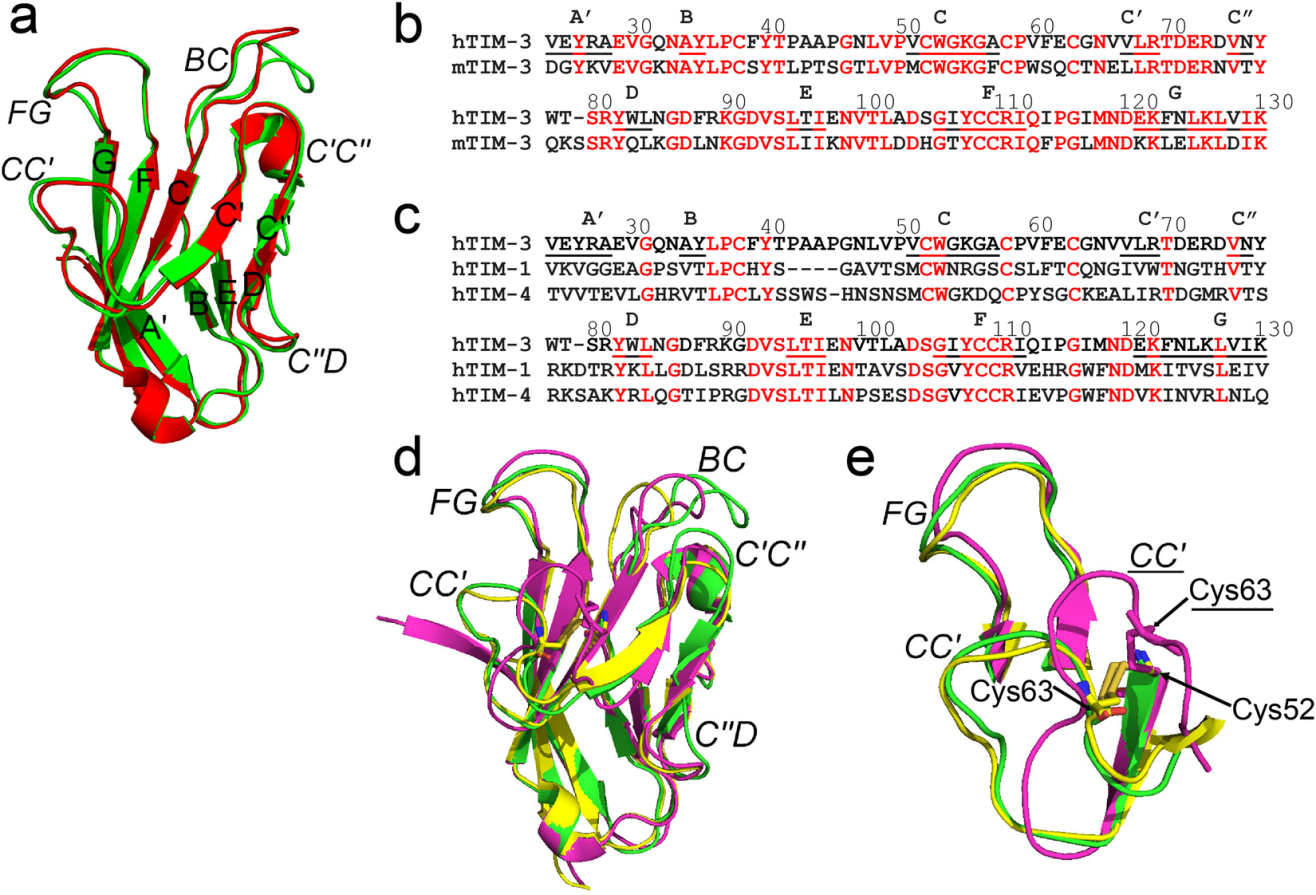
Structural comparison of IgV domains of hTIM-3 with mouse orthologue and other hTIM family members. (**a**) Ribbon diagram of the hTIM-3 (green) and mTIM-3 (red) IgV (PDB code 2OYP) domains superimposed on each other. The β strands are labeled with uppercase letters and loops are highlighted in italics. (**b**) Sequence alignment of the IgV domains of hTIM-3 and mTIM-3. Conserved residues are in red and sequences are numbered. The β strands are labeled and underlined in red and black for conserved and non-conserved β residues, respectively. (**c**) Sequence alignment of the IgV domains of human TIM family members. Conserved residues are in red and sequences are numbered. The β strands are labeled and underlined as above. (**d**) Ribbon diagram of the hTIM-3 (green, PDB code 6DHB), hTIM-1(yellow, PDB code 5DZO), and hTIM-4 (magenta, PDB code 5DZN) IgV domain crystal structures superimposed on each other. Loops are highlighted in italics. (**e**) C-C′ loop conformation differences between human TIM-4 (magenta), hTIM-3 (green) and hTIM-1(yellow). C-C′ (hTIM-1 and hTIM-3) and F-G (hTIM-1, hTIM-3 and hTIM-4) loops are labeled in italics for hTIM family members, whereas C-C′ loop (magenta) for hTIM-4 is labeled in italics and underlined. Residues Cys52 and Cys63 that form noncanonical disulfide bond are aligned in hTIM-3 and hTIM-1, whereas this disulfide bond between Cys52 and Cys63 is significantly deviated in hTIM-4 due to a different CC′ loop conformations and position of Cys63 in hTIM-4. Human TIM-4 CC′ loop and residue Cys63 are underlined. The disulfide bond between Cys52 and Cys63 is shown in orange for hTIM-3 and hTIM-1, and in magenta for hTIM-4.

We next compared the hTIM-3 IgV domain crystal structure with that previously described for mTIM-3 (PDB code 2OYP)^9^. This revealed similarity in the overall structure with a root-mean-square deviation (R.M.S.D) of 0.59 Å (over 599 atoms) (Fig. 2a). For example, the AGFCC′C″ and BED face interactions that are important for stabilization of the IgV fold in mTIM-3 including inter-sheet interactions between Trp53 and Val93, a salt bridge between Arg81 and Asp104 and three disulfide bonds were all conserved in hTIM-3 (Supplementary Fig. 2a). The latter includes disulfide bonds that connect residues Cys38 of the B-C loop and Cys110 of the F strand which provides an anchoring role to stabilize the front and back faces of the hTIM-3 β-sheets and noncanonical disulfide bonds between Cys52 and Cys63, and Cys58 and Cys109. The beta carbon ^13^C NMR chemical shifts of these cysteine residues were observed to be in a range between 38 and 45 ppm, consistent with them being in an oxidized state (average 40.7 +/− 3.8 ppm) through the formation of disulfide bonds rather than in a reduced state (average 28.4+/− 2.4 ppm)^22^ in solution. Similarly, we observed that 33 out of 107 residues (30.9%) were solvent accessible in the hTIM-3 IgV domain compared to 39 solvent accessible residues out of 108 (36.1%) in the mTIM-3 IgV domain.

Despite these similarities, there were significant structural differences between the mTIM-3 and hTIM-3 IgV domains that centered around the B-C, C-C′, C′-C″, and C″-D loops. Notably, the B-C loop of mTIM-3 possesses three proline residues (Pro 37, Pro43 and Pro50) whereas hTIM-3 has an additional proline residue at position 45 (Pro37, Pro42, Pro45 and Pro50). Together with the presence of an additional aromatic residue at position 39 (Phe39) (Fig. 2b), the B-C loop (Ala43-Asn47) of hTIM-3 exhibits substantial changes in its conformation signified by its transposition towards (~4 Å) the α–helix connecting the D and E beta strands (Supplementary Fig. 2b). This involves a carbonyl oxygen atom of hTIM-3 residue Ala44 that makes hydrogen bonded interactions with the main chain amide nitrogen of Asn47. Another important structural difference observed between mTIM-3 and hTIM-3 is evidence for a shorter C″ strand present in hTIM-3 that could impact the conformation of the C″-D loop. In addition, unlike mTIM-3, residues Trp78 and Trp83 of hTIM-3 create a hydrophobic pocket that allows for hydrogen bond formation between the side chain of Thr79 and main chain amide nitrogen of Tyr82. Together, these differences in hTIM-3 result in no apparent outward bending of the C″-D loop in contrast to what is observed in the mTIM-3 structure (Supplementary Fig. 2c). These observed conformational differences in hTIM-3 are likely associated with flexibility in the B-C and C″-D loops regions, account for the missing assignments of the B-C and C″-D loop regions of the molecule in our NMR studies (Supplementary Table 1, Supplementary Fig. 3) and may provide a rationale for the significantly different B-C and C″-D loop conformations observed in a deposited hTIM-3 crystal structure^23^ that lacks bound Ca^++^ (PDB code 5F71).

A characteristic feature of mTIM-3 and the other human TIM family members hTIM-1 and hTIM-4 is the formation of a unique cleft between the C-C′ and F-G loops^2,9–11,21^. This cleft represents a conserved binding site for PtdSer on mTIM-3 as well as hTIM-1 and hTIM-4^2,9,10^. Although the cleft between the C-C′ and F-G loops of human and mouse TIM-3 is generally conserved, hTIM-3 features Val60 and Phe61 residues in the C-C′ loop in contrast to mTIM-3, which has Trp60 and Ser61 residues at these orthologous positions (Fig. 2b). Consequently, we observed a different cleft conformation in hTIM-3 compared to mTIM-3^9^, with an extended distance between the C-C′ and F-G loops in mTIM-3 (~11.5 Å) compared to hTIM-3 (~10.0 Å). Further, the C-C′ loop aromatic residue Trp60 of mTIM3, which has been shown to change the conformation of the mTIM-3 cleft to modulate PtdSer entry and binding, is not conserved in hTIM-3 but is instead replaced by a hydrophobic Val60 residue that lacks an aromatic side chain (Fig. 2b). These structural differences provide distinct cleft volumes of 152.3 Å^3^ for hTIM-3, as shown here, and 50.6Å^3^ for mTIM-3 in the absence^9^ and 191.4 Å^3^ in presence^2^ of PtdSer. This highlights the flexibility of the GF-CC′ cleft volume to accommodate various potential ligands.

### Human TIM family structural comparisons

We also compared the hTIM-3 crystal structure with other human TIM family members^10^ that share >25% sequence identity (Fig. 2c). We found that the IgV domain of hTIM-3 revealed significant structural similarity with hTIM-1 (PDB code 5DZO)^10^ and hTIM-4 (PDB code 5DZN)^10^ as demonstrated by a R.M.S.D of 0.84 Å (over 519 atoms) and 0.92 Å (over 508 atoms), respectively (Fig. 2d). Comparable to the 30.9% solvent accessibility of hTIM-3 IgV domain residues, 32 out of 106 residues (30.2%) and 38 out of 109 residues (34.8%) are solvent accessible in the hTIM-1 and hTIM-4 IgV domains, respectively. Despite these broad similarities, we observed significant conformational differences between hTIM-3 and the other hTIM family members, notably in the B-C, C′-C″ and C″-D loops (Supplementary Fig. 2d). Such differences were also observed in our comparisons of hTIM-3 and mTIM-3 (Supplementary Fig. 2b,c). For example, the B-C loop of hTIM-3 was fourteen residues in length in contrast to hTIM-1, the most restricted human TIM family member, that contained only ten residues. Unlike hTIM-3, the B-C loop of hTIM-1 and hTIM-4 did not exhibit close apposition to an α-helix connecting the D and E beta strands that lies behind the B-C loop. (Supplementary Fig. 2d).

An examination of the hTIM-3 F-G loop in comparison to other TIM family members demonstrated an overall similarity in their conformation. This interestingly occurred despite differences in the primary amino acid sequence. For example, hTIM-3 contains Ile117 and Met118 residues instead of the conserved aromatic residues Trp117 and Phe118 in hTIM-1 and hTIM-4 at these positions which are important for PtdSer binding and lipid bilayer penetration^10^. This observation potentially explains the diminished biochemical affinity that hTIM-3 displays for PtdSer relative to hTIM-1 and hTIM-4^10^. As noted above, the F-G loop of the TIM family members forms a cleft along with the C-C′ loop. It is therefore of interest that the polymorphic Arg111 residue of the hTIM-3 F strand, which is conserved in all human TIM family members, and Lys122 in the hTIM-3 G strand make important hydrogen bond interactions with main chain oxygen atoms of the C-C′ loop residues in hTIM-3.

Of further interest, we observed significant divergence in the C-C′ loop among the human TIM family members. Except for two cysteine residues, most of the C-C′ loop residues are not conserved among the human TIM family members (Fig. 2c). Despite these differences, the overall conformation of the hTIM-3 C-C′ loop is similar to that of hTIM-1^10^. In contrast, we observed that there was a notable conformational difference between the C-C′ loop of hTIM-3 and hTIM-4^10^ in which the C-C′ loop of the latter is observed to rotate inward (Fig. 2e). This is due to the deviation of a noncanonical disulfide bond located between Cys52 and Cys63 of hTIM-4 which confers a distinct C-C′ loop conformation. Consequently, this creates a much larger cleft volume in hTIM-4 (275.5Å^3^) relative to either hTIM-3 (152.3 Å) or hTIM-1 (87.69 Å^3^). Together, this suggests that the GFCC′ face of hTIM-3 is more similar to hTIM-1 than hTIM-4.

### Determination of Ca^++^ binding site of hTIM-3 by x-ray crystallography and NMR that coordinate ligand binding

**Figure 3:**
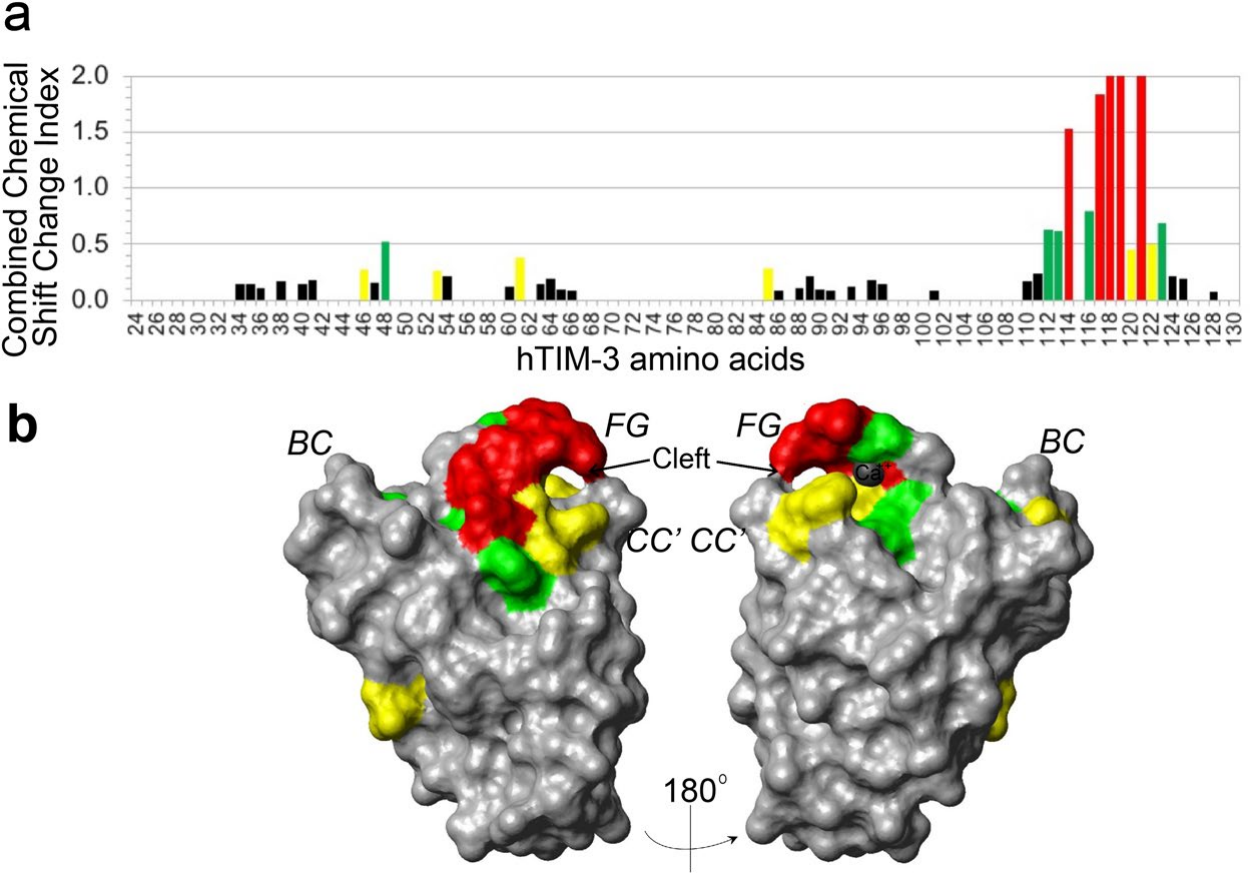
Human TIM-3 IgV domain binding with 10 mM Ca^++^ by NMR. (**a**) Plot of peak shifts showing ^15^N-HSQC combined chemical shift changes index, expressed as [(ΔHcs/0.1 ppm)^2^ + (ΔNcs/0.5 ppm)^2^]^1/2^,of hTIM-3 IgV upon binding with 10 mM Ca^++^. The data columns are colored according to degree of index changes (red > 1.0; green > 0.5; yellow > 0.25). The chemical shift change indices of Met118, Asn119 and Glu121 in F-G loop of hTIM-3 exceeded 2.0, even at well below the saturation concentration of Ca^++^ binding. (**b**) Chemical shift changes of hTIM-3 backbone amides induced by 10 mM Ca^++^ binding, mapped on to hTIM-3 crystal structure surface and colored by degree of index changes as in (**a**). The opposite BED and AGFCC′C″ faces are shown in the left and right panels, respectively, with the B-C, C-C′ and F-G loops marked in italics.

**Figure 4:**
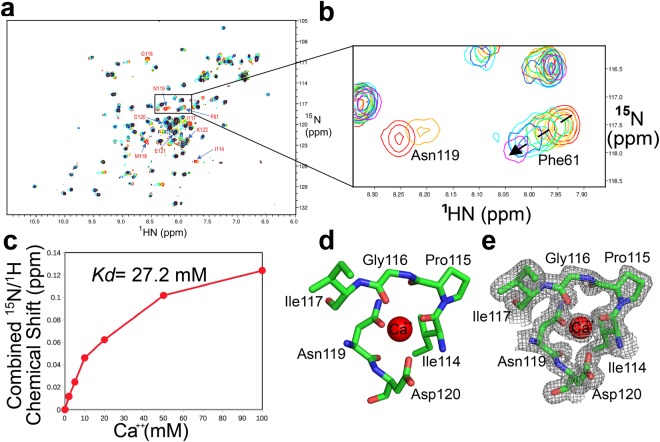
Structural basis of hTIM-3 binding with Ca^++^. (**a**) Overlaid ^15^N-HSQC spectra showing chemical shift changes of hTIM3 backbone amides peaks induced by Ca^++^ binding, from 0 mM (red), 2 mM (orange), 5 mM (yellow), 10 mM (green), 20 mM (cyan), 50 mM (blue) to 100 mM (purple) concentrations. Selected residues of hTIM-3 GFCC′ face (Ile114 to Lys122) that show significant chemical shift changes are labeled. Some of peaks of these residues above 10 mM Ca^++^ concentration are not clear, possibly due to dynamic conformation exchange in solution. (**b**) ^15^N-HSQC peak shift monitoring of the hTIM-3 C-C′ and F-G loop aromatic residues Phe61 (F61) and Asn119 (N119) upon binding with 0–100 mM Ca^++^. (**c**) Determination of Ca^++^ binding constant (Kd) of 27.2 mM for hTIM-3 based on peak shifts of residue Phe61 determined by non-linear regression of the chemical shift change index relative to the Ca^++^concentration. (**d**) Interactions of hTIM-3 F-G loop residues with bound Ca^++^ (red sphere) as observed in the crystal structure. Ile114, Gly116, Asn119, and Asp120 residues of hTIM-3 are highlighted in stick representation and these residues make equal distance coordination (2.3 Å) with Ca^++^. Residues Pro115 and Ile117 of hTIM-3 FG loop are also shown in a stick representation. (**e**) 2Fo-Fc map at 1.0 σ level of hTIM-3 residues Ile114, Pro115, Gly116, Asn119, and Asp120 with bound Ca^++^. Map is superimposed on the final refined model.

The GF-CC′ cleft of mTIM-3, mTIM-4 and hTIM-4 have been defined to possess a metal ion-dependent ligand binding site that is chelated by Ca^++^ and involved in interactions with PtdSer^2,10,19^. Our previously reported NMR studies^4^ have demonstrated that the F-G loop of hTIM-3 exhibits large chemical shift index changes upon the addition of 10 mM Ca^++^ (Fig. 3a,b), and that hTIM-3 binds to hCEACAM1 in the presence of Ca^++ 4^. As shown in Fig. 4a, we performed calcium titration experiments between 0 and 100 mM Ca^++^ and determined a Ca^++^ binding constant (Kd) of 27.2 mM for hTIM-3 by evaluating the peak shifts of the neighboring hTIM-3 C-C′ loop aromatic residue Phe61 through non-linear regression analysis (Fig. 4b,c). Our crystal structure further provides an atomic basis for this interaction and explains the binding mode of Ca^++^ as a cofactor for hTIM-3 function. As observed for other TIM family members, the Ca^++^ binding site of hTIM-3 is located in the F-G loop and involves residues Ile114, Gly116, Asn119, and Asp120 that coordinate a single Ca^++^ cation (Fig. 4d,e, Supplementary Fig. 4), consistent with our NMR findings (Fig. 3a,b). Specifically, the main chain oxygen atoms of Ile114 and Gly116 and side chain oxygen atoms of Asn119 and Asp120 make equidistant (2.3 Å) bonds with the bound Ca^++^ (Fig. 4d,e), leaving two additional sites for ligand-binding coordination. Residues Asn119 and Asp120 that make side chain interactions with Ca^++^ are conserved across all human TIM family members (Fig. 2c). Interestingly, Ile114 is not conserved in hTIM-1, but the main chain oxygen atom of hTIM-1 residue His114 and hTIM-4 residue Val114 are expected to coordinate Ca^++^ similarly to Ile114 in hTIM-3. It is further notable that the hTIM-3 residues Ile114, Asn119, and Asp120 exhibit a very low B-factor consistent with this region providing a highly conserved site in coordinating Ca^++^ (Table 1).

**Figure 5:**
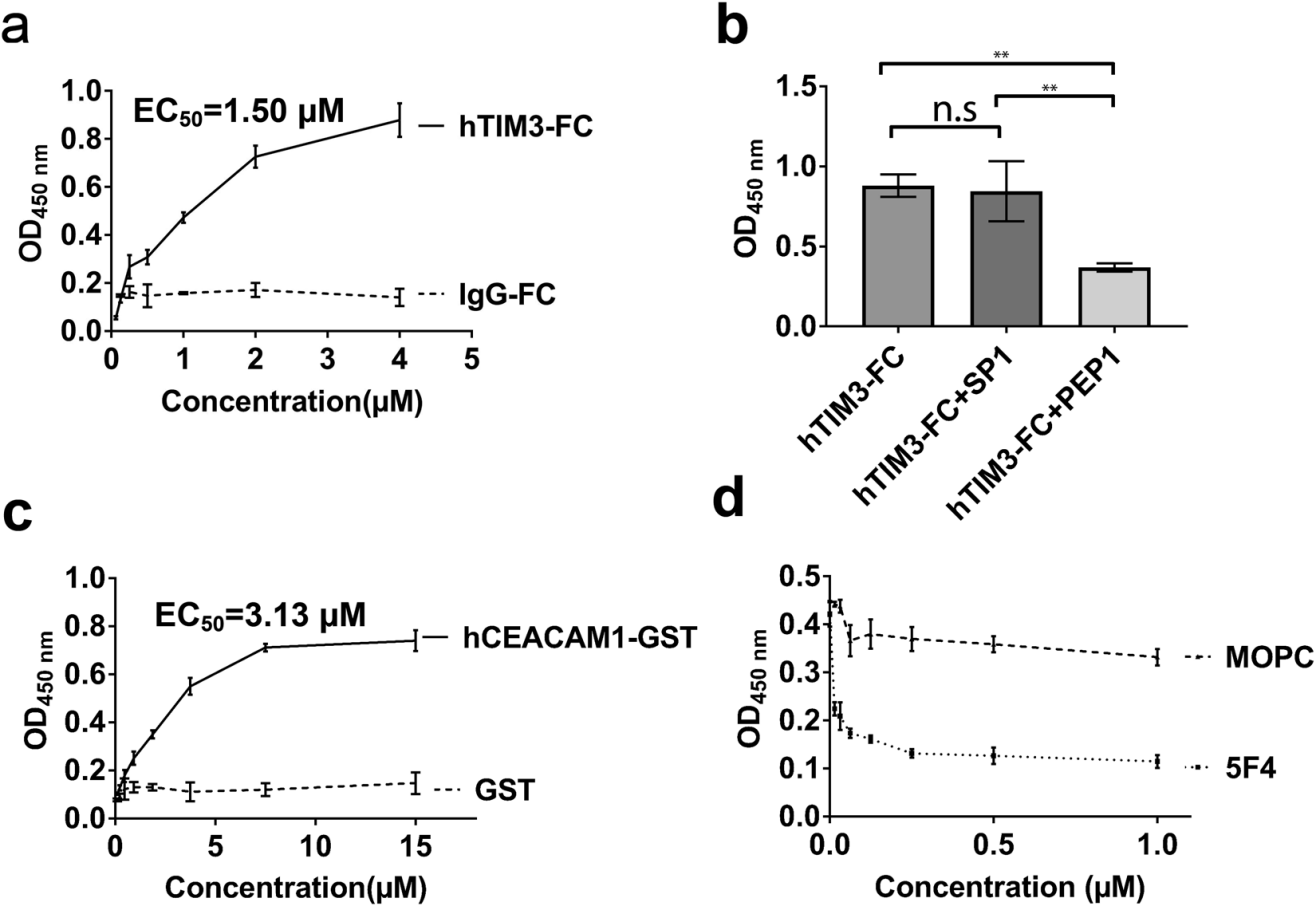
Human TIM-3 and human CEACAM1 binding studies by ELISA. (**a**) hTIM-3 Fc fusion protein but not hIgG-Fc fusion protein (0–4 μM) binds to tagless hCEACAM1 IgV domain with EC_50_ of 1.50 μM. (**b**) Blockade of the interaction between tagless hCEACAM1 and hTIM-3 Fc fusion protein with 10 μM of hTIM-3 C-C′ loop-derived peptide (amino acids 58–77) but not by 10 μM of scrambled peptide. (**c**) Binding of tagless hTIM-3 IgV domain with glutathione-S-transferase (GST) tagged hCEACAM1 IgV-domain protein (0–15 μM) with EC_50_ of 3.13 μM, but not with GST protein (0–15 μM). (**d**) Significant blockade of interaction between tagless hTIM-3 IgV domain and glutathione-S-transferase (GST) tagged hCEACAM1 IgV-domain fusion protein by a mouse anti-human CEACAM1 IgV-domain specific monoclonal 5F4 antibody (0–1 μM), but not by a isotype control MOPC antibody. (**a–d**) ELISA binding assays were performed in triplicate and the average values are shown with standard deviations. n.s., not significant. *p* values ≤ *0.05, **0.01, and ***0.001.

Based on the structural similarity between the human and mouse TIM-3 IgV domains, we also modeled PtdSer at the Ca^++^ binding site of hTIM-3 using the reported co-crystal structure^2^ of PtdSer complexed to mTIM-3 (PDB code 3KAA) as a template. Our model predicts that the hTIM-3 F-G loop residues Asn119 and Asp120 interact with PtdSer through Ca^++^-mediated binding (Supplementary Fig. 5), as reported for other human TIM family members^10,19^. Our model also predicts that the F-G loop residues Ile117 and Met118 residues in hTIM-3 do not form interactions with PtdSer in contrast to residues Trp117 and Phe118 found in hTIM-1 and hTIM-4. Thus, the Ca^++^-bound hTIM-3 crystal structure confirms a conserved metal binding site observed for other TIM family members which is predicted to enable hTIM-3 binding to PtdSer described in previously biochemical studies^10^.

### Biochemical assessment of hTIM-3 binding to hCEACAM1

The well-characterized biochemical assessment and structural studies of hTIM-3 as reported here also allowed us the opportunity to use our previous finding of hTIM-3 binding to hCEACAM1^4^ as a basis to provide evidence that the hTIM-3 protein studied here is functional. We therefore established an enzyme-linked immunosorbent assay (ELISA) assay to evaluate hCEACAM1-hTIM-3 interactions. First, we observed binding of a hTIM-3 Fc fusion protein in solution to plate-bound tagless hCEACAM1 IgV domain, as detected by an anti-human IgG Fc specific horseradish peroxidase conjugated antibody (Fig. 5a). Further, this interaction was blocked by a hTIM-3 C-C′ loop-derived peptide (amino acids 58–77) but unperturbed by a scrambled control peptide (Fig. 5b). Consistent with our previously reported hTIM-3 mutagenesis studies^4^, this hTIM-3 C-C′ loop-derived peptide possesses h-TIM-3 residues Glu62 and Arg69 which are critical for hCEACAM1 binding. In an alternative approach, we applied the tagless hTIM-3 IgV domain used in crystallization experiments to a plate and observed binding of a glutathione-S-transferase (GST) tagged hCEACAM1 IgV-domain protein, but not GST alone (Fig. 5c). The latter interaction was blocked by a mouse anti-human CEACAM1 IgV-domain specific monoclonal antibody (5F4)^24^, but not by an isotype control antibody (Fig. 5d). Together, these studies show that the hTIM-3 IgV domain protein used in the structural studies exhibit functional binding activity.

## Discussion

**Figure 6:**
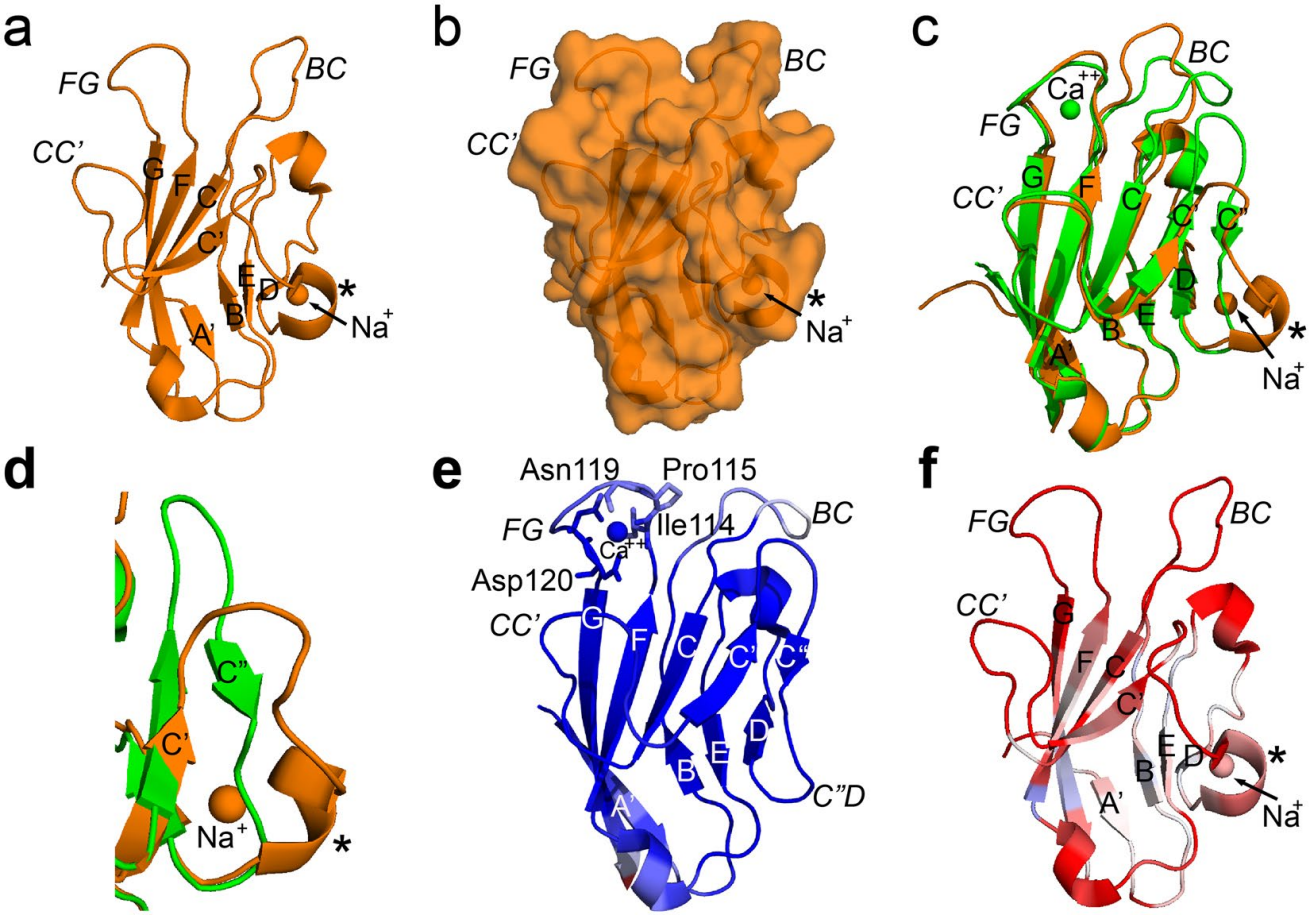
Ca^++^-bound hTIM-3 structural comparison (PDB code 6DHB) with Na^+^-bound hTIM-3 (PDB code 5F71). An α- helix that appears only in the Na^+^-bound hTIM-3 structure instead of C″ strand is marked by an asterisk (*). The β strands are labeled with uppercase letters and loops are highlighted in italics. (**a**) Ribbon diagram of the 2.4 Å resolution crystal structure of hTIM-3 with bound Na^+^. (**b**) Surface view of Na^+^-bound hTIM-3. (**c**) Structural alignment between Ca^++^-bound hTIM-3 crystal structure (green, PDB code 6DHB) with Na^+^-bound hTIM-3 crystal structure (orange, PDB code 5F71). (**d**) The lack of a C″ strand in the Na^+^-bound hTIM-3 crystal structure (orange) and appearance of an α- helix (marked by an asterisk) at this region. Ca^++^-bound hTIM-3 is shown in green and C″ strand as observed in Ca^++^-bound hTIM-3 crystal structure is labeled. (**e**) Ribbon diagram of hTIM-3 crystal structure with bound Ca^++^. The loops, strand, residues and Ca^++^ are colored based on B-factor range (blue-white-red, where blue minimum = 10, red maximum = 40). (**f**) Ribbon diagram of Na^+^-bound hTIM-3 crystal structure. The loops, strand, residues and Na^+^ are colored based on B-factor range (blue-white-red, where blue minimum = 10, red maximum = 40).

We have performed a biophysical and structural assessment of the hTIM-3 IgV domain. To do so, we performed the first NMR analysis of a TIM family member and solved a high resolution (1.7 Å). crystal structure of hTIM-3 IgV domain. This has allowed us the opportunity to decipher the structural details of hTIM-3 at an atomic level. Since the description of the first crystal structure for mTIM-3 (PDB code 2OYP)^9^ in 2007, there is only one report of a low resolution (2.4 Å) hTIM-3 structure (Fig. 6a) described in the literature (PDB code 5F71)^23^. This crystal structure of hTIM-3 exhibited several structural features that are worthy of mention relative to the crystal structure described here. Notably, the previously reported crystal structure lacks a fully formed channel between the C-C′ and F-G loops of hTIM-3 structure (Fig. 6b), as observed in mTIM-3^9^ and our hTIM-3 crystal structure reported here (Fig. 1c). In addition, this hTIM-3 crystal structure (PDB code 5F71) exhibits significant conformational differences in the B-C, C′-C″, and C″-D loops. (Fig. 6c). More specifically, this previously reported hTIM-3 structure completely lacks a C″ beta strand and instead contains an additional alpha helix with bound sodium (Na^+^) in this location (Fig. 6d). This additional helix with bound sodium was not observed in mTIM-3^9^ and our hTIM-3 crystal structure. The finding of Na^+^ in PDB code 5F71 is perhaps consistent with the presence of sodium abundant crystallization conditions^23^. These overall structural characteristics are also associated with a high average B factor for all atoms (40 Å^2^)^23^, as judged by the PDB validation report (Fig. 6f). This is in contrast to our hTIM-3 crystal structure that possesses a very low average B factor for all atoms (13 Å^2^) and no PDB validation outliers (side-chain, Ramachandran and RSRZ) as judged by the PDB validation report (Fig. 6e). Most importantly, our structure, but not PDB code 5F71^23^, is Ca^++^-bound to amino acids in the GFCC′ face. This is particularly important as numerous studies^2,10,19^ consider Ca^++^ to be an essential factor for TIM-family binding to its ligands suggesting our structure is representative of the biologically active state of hTIM-3. Thus, the hTIM-3 crystal structure reported here most likely resembles the *in vivo* biologically relevant Ca^++^-bound state of this important immunologically active molecule.

These studies also provide important insights into the mechanism by which the hTIM-3 GFCC′ face may bind its various ligands and highlights the similarities and conformational differences of hTIM-3 relative to other hTIM family members and mTIM-3. It is speculated that the presence of these significant structural differences between mTIM-3 and hTIM-3 may impact their species-specific functions. Moreover, using the hTIM-3 IgV protein used in the structural studies described, we established a novel ELISA assay to assess hTIM-3 functionality through its ability to bind hCEACAM1 as previously shown^4^. In addition, we showed that this interaction was blocked by a peptide derived from the hTIM-3 C-C′ loop that contained amino acid residues previously shown be involved in hCEACAM1 interactions^4^ and a mouse anti-human CEACAM1 antibody specific for the CEACAM1 N-domain^24^. These latter studies further emphasize the importance of the GFCC′ face of hTIM-3 in ligand binding and especially those that involve hCEACAM1.

Several polymorphisms in mouse and human TIM-3 have been previously identified and shown to be associated with alterations of ligand interactions and/or disease susceptibility^4,9,25^. Our structural studies provide potential insights into these observations. For example, polymorphisms in the mTIM-3 B-C loop at the Pro43, Ser45 and Thr47 residues have been described that are associated with autoimmune and allergic disease susceptibility. Specifically, the allergy-susceptible BALB/c mouse strain possesses a Ser45 residue at this position in contrast to the DBA/2 mouse strain that includes Pro45 and exhibits resistance to allergy in models of airway hypersensitivity^9,25^. Interestingly, hTIM-3 exhibits an orthologous Pro45 residue at this position that results in an extra proline residing in the B-C loop of hTIM-3 compared to mTIM-3 and causes conformational changes in the B-C loop as described herein (Supplementary Fig. 2b). On the other hand, polymorphisms in hTIM-3 including Cys58Arg (rs201750016) and Arg111Trp (rs145478313), which would be predicted to disrupt the GF-CC′ cleft based upon our crystal structure, have been shown to affect the biochemical interactions between hTIM-3 and hCEACAM1^4^.

**Figure 7:**
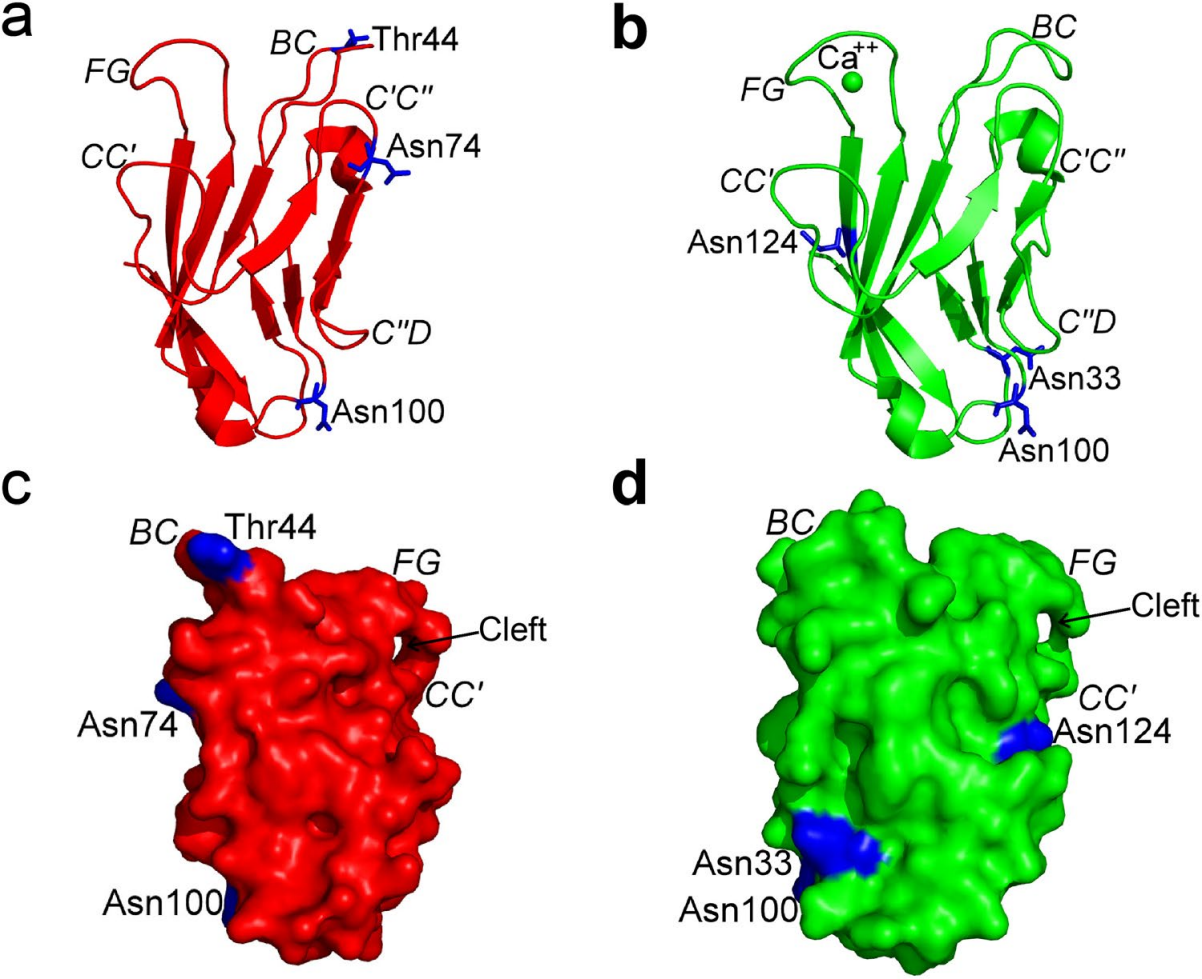
Comparison of potential glycosylation sites of hTIM-3 and mTIM-3. (**a**) Potential N- and O-glycans sites of mTIM-3 mapped at residues Thr44, Asn74, and Asn100. mTIM-3 crystal structure (PDB code 2OYP) is shown in red ribbon representation and potential glycosylation sites are highlighted in blue stick representation. (**b**) Potential N- and O-glycans sites of hTIM-3 mapped at residues Asn33, Asn100, and Asn124. hTIM-3 crystal structure is shown in green ribbon representation with bound Ca^++^ and potential glycosylation sites are highlighted in blue stick representation. (**c**) Surface view of mTIM-3 glycan sites in blue. The mTIM-3 molecule is rotated ~180^°^ about a vertical axis when compared to mTIM-3 molecule as shown in (**a**). (**d**) Surface view of hTIM-3 glycan sites in blue. The hTIM-3 molecule is rotated ~180^°^ about a vertical axis when compared to hTIM-3 molecule as shown in (**b**).

The high resolution of our hTIM-3 crystal structure also allowed mapping of potential glycosylation sites on hTIM-3, which is of interest since TIM-3 interacts with galectin-9 through the glycans on the TIM-3 IgV domain^1,9^. In comparison to the glycosylation sites of mTIM-3 that map to Thr44 (B-C loop), Asn74 (C′-C″ loop) and Asn100 (E-F loop) residues, the glycosylation sites of hTIM-3 are predicted to exist at residue Asn33 (A′-B loop), Asn100 (E-F loop) and Asn124 (G strand) (Fig. 7a–d). It is notable that while all three glycosylation sites for mTIM-3 are located at opposite side of the GFCC′ face^9^ (Fig. 7a,c), the localization of the glycosylation sites of hTIM-3 were distinctly different. Specifically, the glycosylation site at residue Asn124 of hTIM-3 is present on the G strand which causes it to lie adjacent to the GF-CC′ cleft and, interestingly, the glycosylation sites at Asn33 and Asn100 that create a continuous glycan surface on hTIM-3 where galectin-9 binds based on studies with mTIM-3 (Fig. 7b,d). Together, these differences may predict unique interactions of galectin-9 with hTIM-3 relative to mTIM-3 and the possibility that carbohydrate side-chain modifications at Asn124 may affect hTIM-3 interactions with ligands that bind to the GFCC′ face.

Recent studies have suggested that therapeutic agents capable of exhibiting optimal anti-tumor responses target hTIM-3 on the GFCC′ face where most known hTIM-3 ligands bind^5^. Our structural studies of hTIM-3 also allow for a deeper understanding of these targeting agents. An example of this is the anti-hTIM-3 antibody 2E2 that has immunomodulatory properties and binds to the CC′ and FG loops of the hTIM-3 IgV^4,5^ but not hTIM-1 IgV domain^13^ (Supplementary Fig. 6a). Consistent with this, peptide competition assays show that a hTIM-3 C-C′ loop-derived peptide, but not a scrambled control peptide, inhibits binding of mouse anti-human 2E2 antibody to hTIM-3 expressed on exhausted CD4^+^ T-cells or transfected Jurkat T cells^17^ (Supplementary Fig. 6b). Our studies provide a potential explanation for this selectivity which may be useful in designing future therapeutic agents to specifically target hTIM-3. Interestingly, our structural comparisons of hTIM-1 and hTIM-3 show that hTIM-1, despite exhibiting a similar conformation of the GFCC′ face, has non-conserved aromatic residues Trp117 and Phe118 at the tip of the hTIM-1 FG loop (Supplementary Fig. 6c). It is predicted that these residues may impose direct steric hindrance on therapeutic antibody binding to hTIM-1, as we predict is the case for the 2E2 antibody.

In conclusion, the high-resolution crystal structure and NMR analysis described herein provide structural mapping of the hTIM-3 IgV domain in the context of its various roles. It is anticipated that these studies will guide future understanding of these interactions and the development of novel therapeutic agents directed at hTIM-3 and those that are currently in human clinical development.

## Methods

### Protein purification, Crystallization, data collection, structure determination and analysis

Human TIM-3 IgV domain protein (N-terminal methionine followed by Val24 thru Lys130) was purified using our previously published protocols with slight modifications in refolding conditions^4^. Briefly, refolding of human TIM-3 IgV was performed by drop-wise addition of solubilized inclusion bodies into 1 liter of a refolding buffer consist of 100 mM Tris pH 8.3, 0.4 M L-arginine, 2 mM EDTA, 5 mM reduced GSH and 0.5 mM oxidized GSH and refolding mixture was stirred overnight at 4 °C. Next, the mixture was concentrated to 100 ml and dialyzed three times against a dialysis buffer containing 20 mM Tris pH 7.5, 200 mM NaCl, and 1 mM EDTA. The dialysate was concentrated to 2.5 ml and hTIM-3 was isolated by size exclusion chromatography on a HiPrep 16/60 Sephacryl S-200 HR column (GE Healthcare) in running buffer containing 10 mM HEPES 7.5, 100 mM NaCl. A single peak for human TIM-3 IgV domain was collected and the protein was concentrated to 10 mg/ml supplemented with 10 mM CaCl_2_ for crystallization. Tagless hCEACAM1 IgV and GST-hCEACAM1 IgV fusion proteins were expressed and purified as described^4^.

The initial crystal screenings were performed in a Intelli-Plate 24-well sitting-drop format (Art Robbins) with Index Screens 1 and 2 (Hampton Research, USA) at room and 4 °C temperature. Promising conditions were further optimized by varying the protein: precipitant ratio around initial hits. Crystals which were later used for X-ray diffraction experiments for a Ca^++^ -bound hTIM-3 crystal structure were formed above in a 250 μL solution volume containing 0.1 M Tris pH 8.5, 25% w/v Polyethylene glycol 3,350 at room temperature for 10 days. For data collection, crystals were cryoprotected in a solution containing glycerol, glucose solution in aqueous benzoic acid, ethylene glycol, and sucrose. Although we screened numerous hTIM-3 protein crystals from at the LS-CAT beamlines at Advanced Photon Source (APS; Argonne, IL, USA) and National Synchrotron Light Source- II(NSLS-II, Upton, NY, USA) 17-ID beamlines, the high resolution X-ray data that was used to solve the Ca^++^-bound hTIM-3 structure were collected using the LS-CAT beamlines 21-ID-F/G at Advanced Photon Source (APS; Argonne, IL, USA). The data was processed with the HKL2000^26^, iMosflm^27^ and the CCP4 suite^28^. During the data collection, the initial space group was determined with HKL2000 software which was available to us at the beamline sources (APS, Argonne, IL; NSLS-II, Upton, NY). Once the initial data collection was completed, we determined the space group of the whole data set with iMosflm software which was easily available to us in our laboratory. During the data processing, we set the high-resolution limit at 1.7 Å based upon the overall and outer shell completeness. Although the I/sigma (I) value was 10.5 in the highest resolution shell, when we increased the highest resolution shell to 1.6 Å or further, we observed a considerable decrease in completeness of the highest resolution shell. As a result, we embraced a conservative approach and limited the highest resolution shell to 1.7 Å.

The structure of the hTIM-3 IgV domain with Ca^++^ was determined by a molecular replacement method using a modified N-terminal domain of a another hTIM-3 crystal structure (PDB code 5F71)^23^ as a search model, with all hTIM-3 amino acids changed to alanine to reduce model bias and all ligands, water and metal ions, were removed. Molecular replacement was performed with Molrep, refinements were performed with the Refmac and intermittent model building was performed with COOT^29^. 5% of the total reflection data was excluded from the refinement cycles and used to calculate the free Rfactor (*R*_*free*_) for monitoring the refinement progress. We used 10 cycles of maximum likelihood Restrained refinement using isotropic B factors with “Use automatic weighing″ option turned on. Although some of loops and strands fitted properly during the early refinement process with *R*_*work*_/*R*_*free*_ of 42/45%, significant structural remodeling of entire B-C, C-C′, C′-C″, C″-D and F-G loops, guided by a mTIM-3 crystal structure^9^ (PDB code 2OYP), were required for better fitting and refinement. An electron density for a metal ion consistent with Ca^++^ was identified in the crystal structure after rigid body and subsequent refinements associated with model building. Notably, Ca^++^ was fitted with an occupany of 1 only after fitting of all hTIM-3 residues, which led to the final crystallographic *R*_*work*_/*R*_*free*_ of 15%/18% at 1.7 Å resolution for a Ca^++^ bound hTIM-3 structure. An electron density associated with benzoic acid was noted adjacent to the Ca^++^ binding site that was fit during the refinement. We also tried to fit sodium (Na^+^) instead of Ca^++^ for fitting a metal ion density as described above, but an improved structural model, electron density maps and *R*_*work*_/*R*_*free*_ were obtained with Ca^++^ that included interactions with residues of the hTIM-3 F-G loop similar to that observed in our NMR binding experiments. While Rfactor/Rfree values of 0.1502/0.1805 were observed for Ca^++^, higher Rfactor/Rfree values of 0.1521/0.1812 were observed for Na^+^, with a significant positive Fo-Fc map density, supporting Ca^++^ as the observed metal ion. Given that Ca^++^ exhibits a significant anomalous signal in contrast to Na^+^, we used this information to create anomalous difference Fourier (using CCP4 FFT)^28^ and LLG (using Phaser-EP “ MR-SAD phasing″ gui in PHENIX)^38^ maps. These maps clearly revealed the peak as representative of Ca^++^ even at 5 sigma. Thus, we concluded that Ca^++^ exhibited a better fit which was consistent with the interactions observed in our NMR studies. The atomic coordinates and structure factors for the Ca^++^ bound hTIM-3 crystal structure were deposited with RCSB accession code 6DHB.

The X-ray data and structure refinement statistics are shown in Table 1. All the figures were drawn using PyMOL (DeLano Scientific); sequence alignments were done using Clustal Omega^30^, solvent accessible residues of Ca^++^-bound hTIM-3 crystal structure reported here were determined by Swiss-PDB viewer using 30% accessibility of residues by solvent; cavity volume (Connolly’s molecular surface) between C-C′ and F-G loop was determined using CASTp webserver^31^, and mapping of glycosylation sites was done using GPP prediction webserver^32^.

### Differential scanning fluorimetry

Differential scanning fluorimetry experiments were performed on a QuantStudio 6 (Applied Biosystems). Samples of hTIM-3 IgV domain (20 µM) were prepared with 0–10 mM CaCl_2_ and incubated for 4 hours at 4 °C. 20 µL reactions were set up in a 96 well PCR plate comprised of each hTIM-3/CaCl_2_ mixture, 1000x fluorescent probe (Protein Thermal Shift Dye Kit, ThermoFisher) in triplicate and subjected to heat ramp from 25–99 °C. Fluorescence was measured in 0.25 °C intervals and the melting temperatures (T_m_) were calculated from the respective fluorescence profiles using the Boltzmann method in Protein Thermal Shift™ Software (Applied Biosystems). Standard error was calculated from experimental triplicate sample repeats.

### NMR assignments of human TIM3 IgV domain

^15^N and ^13^C double-labeled hTIM-3 IgV domain protein were expressed from *E. coli* in M9 minimal medium containing ^15^NH_4_Cl and ^13^C-gluocse as the sole nitrogen and carbon sources, and purified as described above. Non-uniformly-sampled (NUS) triple resonance experiments using ^15^N/^13^C-hTIM-3 IgV (0.85 mM) in 10 mM HEPES, 50 mM NaCl, pH 7.0 with 10% D_2_O, were performed at 25 °C on a 700 MHz Agilent DD2 spectrometer equipped with a cryogenic probe. The data were processed using NMRPipe^33^ and Iterative Soft Thresholding reconstruction approach (istHMS)^34^ and analyzed by CARA^35^. Overall 75% of backbone amide resonances were assigned, along with CO, CA and CB ^13^C resonances. The missing assignments in the C′, C″ and D strand regions are likely caused by conformation exchange dynamics in solution in the absence of human TIM3 ligands. Secondary structure predications based on assigned chemical shifts were obtained using the TALOS+ software^36^. The NMR data and assigned chemical shifts were deposited in the BMRB database with accession code 27525.

### NMR titration study of human TIM3 IgV domain

Calcium titration experiments were performed using 0.05 mM ^15^N-hTIM-3 IgV domain purified protein titrated with 0 mM to 100 mM CaCl_2_ on a 600 MHz Agilent DD2 spectrometer at 25 °C, processed by NMRPipe^33^ and analyzed by CARA^35^. An initial estimate of the dissociation constant of Ca^++^ binding to the un-ligated hTIM-3 IgV domain yielded K_D_ of 22 mM by fitting an approximate linear solution to the reciprocal plot of the Phe61 chemical shift change relative to the Ca^++^ concentration. More rigorous non-linear regression analysis of the dissociation constants were further performed using Microsoft Excel by fitting the chemical shift change index δ = sqrt((ΔNcs/5)^2^ + (ΔHcs)^2^) relative to the Ca^++^ concentration according to the equation provided by Morton *et al*.^37^. By non-linear regression analysis, the average dissociation constant of Ca^++^ binding to the un-ligated hTIM-3 IgV was determined as 27.8 mM for five amide peaks with the largest observable shift changes being 26.6 mM, 27.2 mM, 24.6 mM, 29.5 mM and 31.0 mM for Leu48, Phe61, Arg89, Arg111 and Gln113 respectively.

### ELISA binding and blockade experiments

ELISA binding studies were performed using Costar 96-well high protein binding plates (Corning). For binding studies between human TIM-3 Fc fusion protein (BPS Bioscience) and tagless hCEACAM1 IgV domain, 50 μl of 250 nM tagless hCEACAM1 protein in Tris-buffered saline buffer containing 10 mM CaCl_2_ (TBS-Ca^++^) or TBS-Ca^++^ buffer alone was added to wells of an ELISA plate and incubated overnight at 4 °C. After incubation overnight, the wells were washed three times with TBS buffer containing 0.05% Tween 20 and 10 mM CaCl_2_ (TBST-Ca^++^), blocked with 2% BSA in TBS-Ca^++^ buffer for 1 hour at room temperature, and incubated with hTIM-3 Fc fusion protein (0–4 μM) or hIg-Fc fragment protein (Abcam) in TBS-Ca^++^ buffer for two hours at 37 °C. The wells were washed three times with TBST-Ca^++^ buffer and incubated with goat anti-human IgFc-HRP conjugated antibody (Southern Biotech) for 1 hour at room temperature. The wells were washed three times with TBST-Ca^++^ buffer and treated with TMB peroxidase substrate solution (Seracare). The reaction was stopped using 3 M HCl and OD values were obtained at 450 nm. Data were plotted using GraphPad Prism software and EC_50_ was determined. Similar ELISA protocols were used for peptide blocking experiments where binding of tagless hCEACAM1 was measured with hTIM-3 Fc fusion protein (4 μM) incubated with 10 μM of hTIM-3 C-C′ loop-derived peptide (amino acids 58–77, 58-CPVFECGNVVLRTDERDVNY-77)) or 10 μM of scrambled control peptide (TLCVCFVNPYDVRVNDEREG). The peptides were synthesized by American Peptide Company, CA and with > 98% purity. Data were plotted using GraphPad Prism software and *p* values determined using 1 way Anova-Tukey’s multiple comparisons test, where the mean of each binding column was compared with the mean of every binding column. For ELISA binding experiments between tagless human TIM-3 protein used for structural studies and GST tagged hCEACAM1 IgV-domain protein, 50 μl of 250 nM tagless hTIM-3 protein was added to wells of ELISA plates using similar ELISA protocols described above in Dulbeccos PBS buffer (Sigma Aldrich) and binding of GST tagged hCEACAM1 IgV domain protein (0–15 μM), or GST control protein (Sigma Aldrich), was detected using goat polyclonal anti-GST-HRP conjugated antibody (Abcam). Binding data were plotted and EC_50_ was determined in Graphpad Prism. Blockade of tagless hTIM-3 binding to GST tagged CEACAM1 IgV domain protein was performed in the presence of a mouse anti-human CEACAM1 IgV domain specific monoclonal, 5F4 (0–1 μM), or mouse IgG1 isotype control antibody, using 4 μM GST-tagged hCEACAM1 IgV domain protein.

### Peptide competition assays

For *in vitro* CD4^+^ T cell activation to induce exhaustion, MACS purified human CD4^+^ T cells were cultured in complete medium RPMI 1640 (Lonza) supplemented with 10% fetal calf serum (FCS), 1% glutamine, 100 IU/ml penicillin, 100 μg/ml streptomycin (Life Technologies), 25 mM HEPES (Sigma-Aldrich) in 96-well plates at a concentration of 40 IU/ml recombinant (r)IL-2 (NIH) and soluble anti-CD3 (1 μg/ml) in a total volume of 200 ml. Cell cultures were refreshed with new rIL-2 and new soluble anti-CD3 every 5 days. After three rounds of stimulation, exhausted CD4^+^ T cells were generated. Jurkat hTIM-3 transfected Jurkat T cells have been previously described^17^. The samples from the exhausted CD4^+^T cells and Jurkat hTIM-3 transfectants were solubilized by suspension in Laemmli sample buffer and the proteins resolved by SDS–PAGE under reducing conditions followed by transfer to a PVDF (polyvinylidene difluoride) membrane. After blocking with 5% of skim milk in 0.05% PBS-Tween (PBS-T) buffer, the membranes were incubated for 16 hr at 4 °C with mouse anti-human 2E2 antibody in the presence of 15-fold molar excess of a hTIM-3 C-C′ loop-derived peptide or scrambled control peptide. The membranes were incubated with goat anti-mouse IgG HRP conjugated antibody (Southern Biotech) for 1 hr at room temperature and visualized by Amersham ECL Western Blotting Detection Reagents (GE Healthcare).

## Electronic supplementary material

Supplementary Information
