# Supplementary material for: High resolution X-ray and NMR structural study of human T-cell immunoglobulin and mucin domain containing protein-3
Source: Sci Rep. 2018 Nov 30;8:17512. doi: 10.1038/s41598-018-35754-0 (PMC6269442; doi:10.1038/s41598-018-35754-0)
Supplement: Supplementary file 1 — Supplementary Information [file 41598_2018_35754_MOESM1_ESM.pdf]

High resolution X-ray and NMR structural study of human T-cell immunoglobulin and mucin domain containing protein-3

Amit K. Gandhi[1,10], Walter M. Kim[1,10], Zhen-Yu J. Sun[2,6,10], Yu-Hwa Huang[1], Daniel A. Bonsor[3], Eric J. Sundberg[3,7,8], Yasuyuki Kondo[1,9], Gerhard Wagner[2], Vijay K. Kuchroo[4], Gregory Petsko[5], Richard S. Blumberg[1,*]

[1] Division of Gastroenterology, Department of Medicine, Brigham and Women's Hospital, Harvard Medical School, 75 Francis Street, Boston, MA 02115, USA.
[2] Department of Biological Chemistry and Molecular Pharmacology, Harvard Medical School, 240 Longwood Avenue, MA 02115, USA.
[3] Institute of Human Virology, University of Maryland School of Medicine, University of Maryland, 725 W Lombard St, Baltimore, MD 21201,USA.
[4] Evergrande Center for Immunologic Diseases and Ann Romney Center for Neurologic Diseases, Harvard Medical School and Brigham and Women's Hospital, 77 Avenue Louis Pasteur, Boston, MA 02115, USA.
[5] Department of Neurology and Feil Family Brain and Mind Research Institute, Weill Cornell Medical College, New York, NY 10021, USA.
[6] Current address: Department of Cancer Biology, Dana-Farber Cancer Institute, Boston, MA 02215, USA
[7] Department of Medicine, University of Maryland School of Medicine, University of Maryland, Baltimore, MD 21201,USA.

[8] Department of Microbiology and Immunology, University of Maryland School of Medicine, University of Maryland, Baltimore, MD 21201,USA.
[9] Current address: Division of Gastroenterology, Department of Internal Medicine, Graduate School of Medicine, Kobe University, Kobe, 650-0017, Japan.
[10] These authors contributed equally to this work.
* To whom correspondence should be addressed: rblumberg@bwh.harvard.edu.

Supplementary Figure 1

a

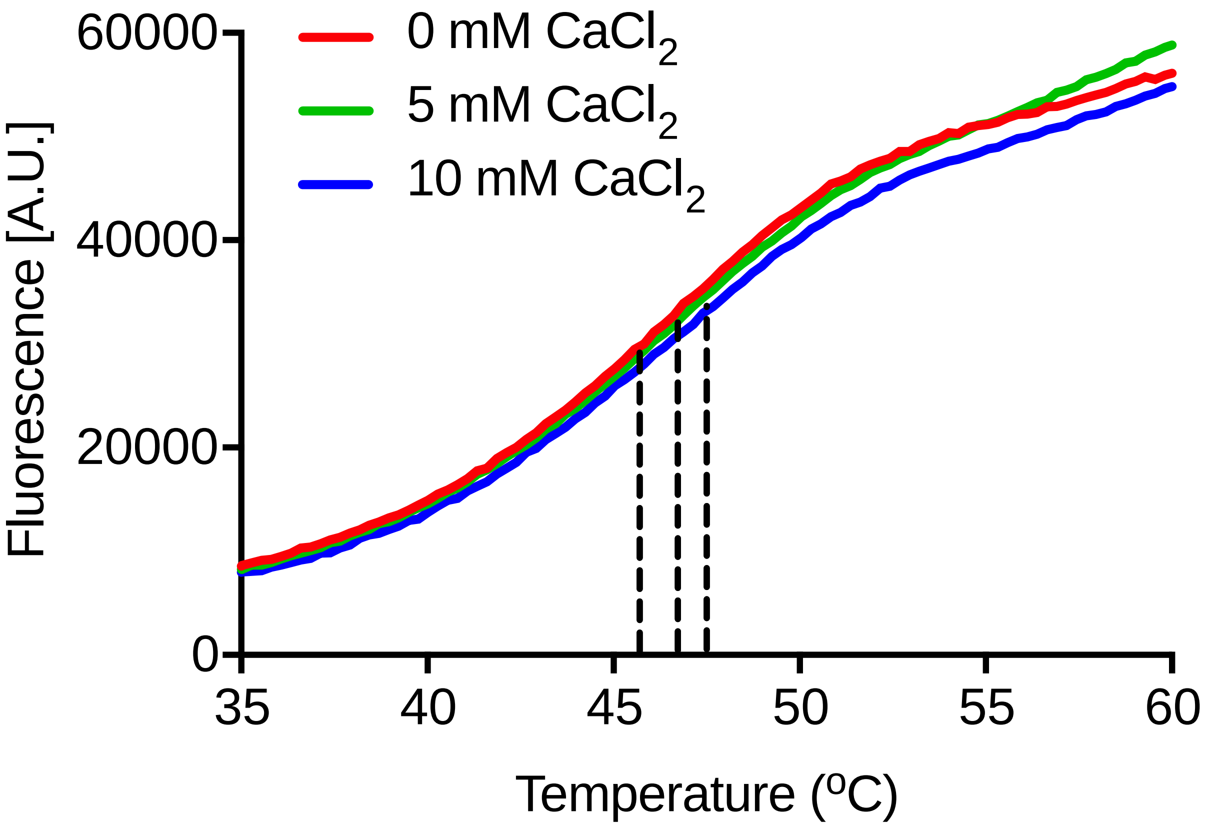

b

**Additive**	**Tm (°C) [Std Error]**
0 mM $CaCl_2$	45.72 [± 0.200]
5 mM $CaCl_2$	46.71 [± 0.104]
10 mM $CaCl_2$	47.51 [± 0.180]

Supplementary Figure 2

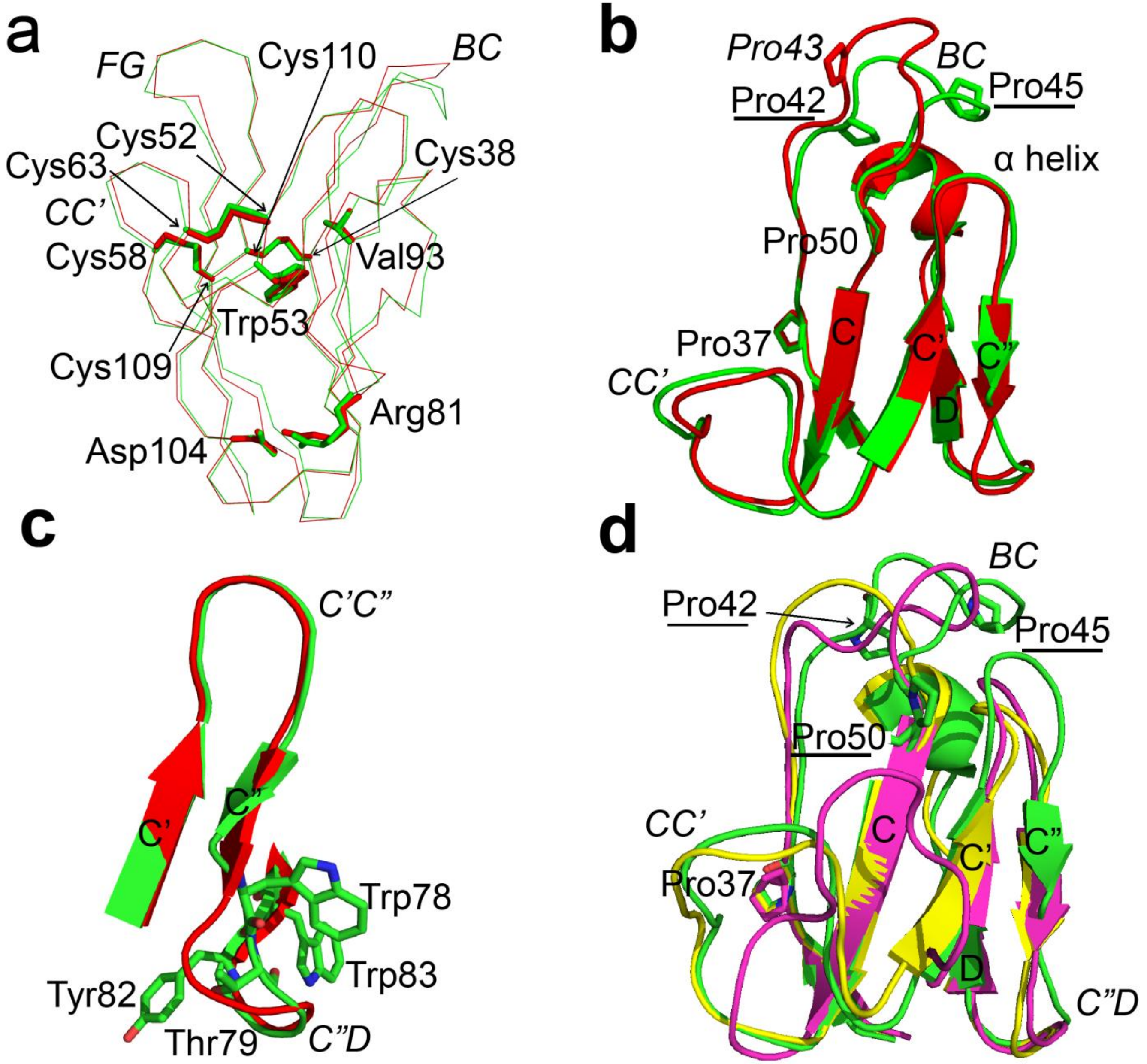

Supplementary Figure 3

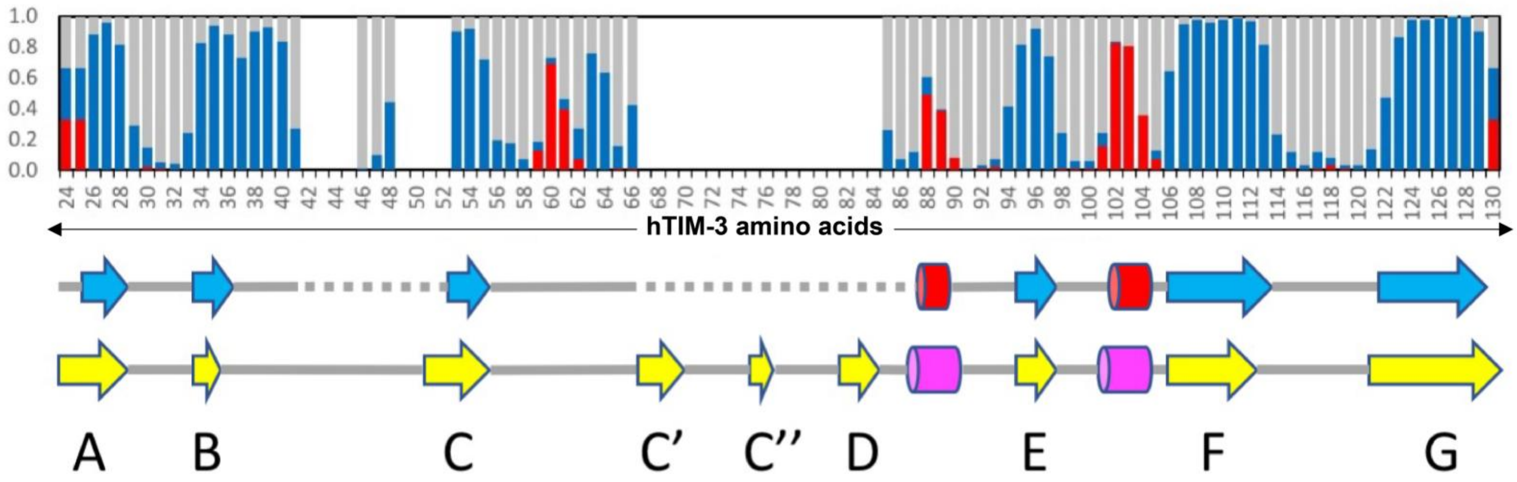

Supplementary Figure 4

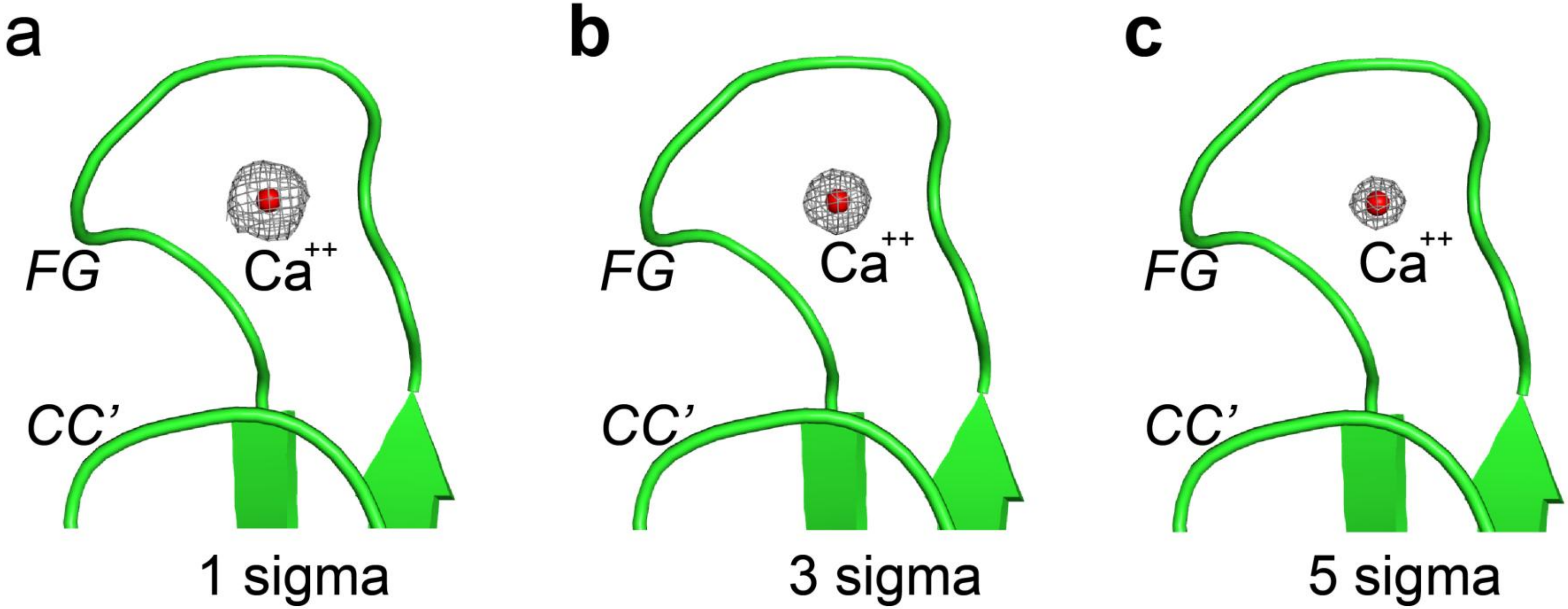

Supplementary Figure 5

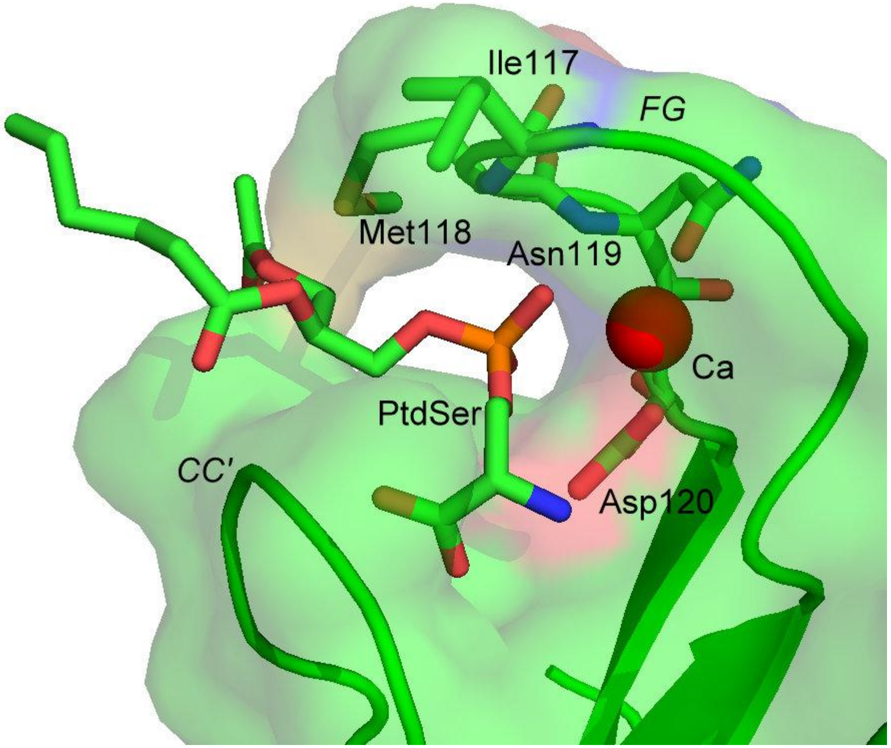

Supplementary Figure 6

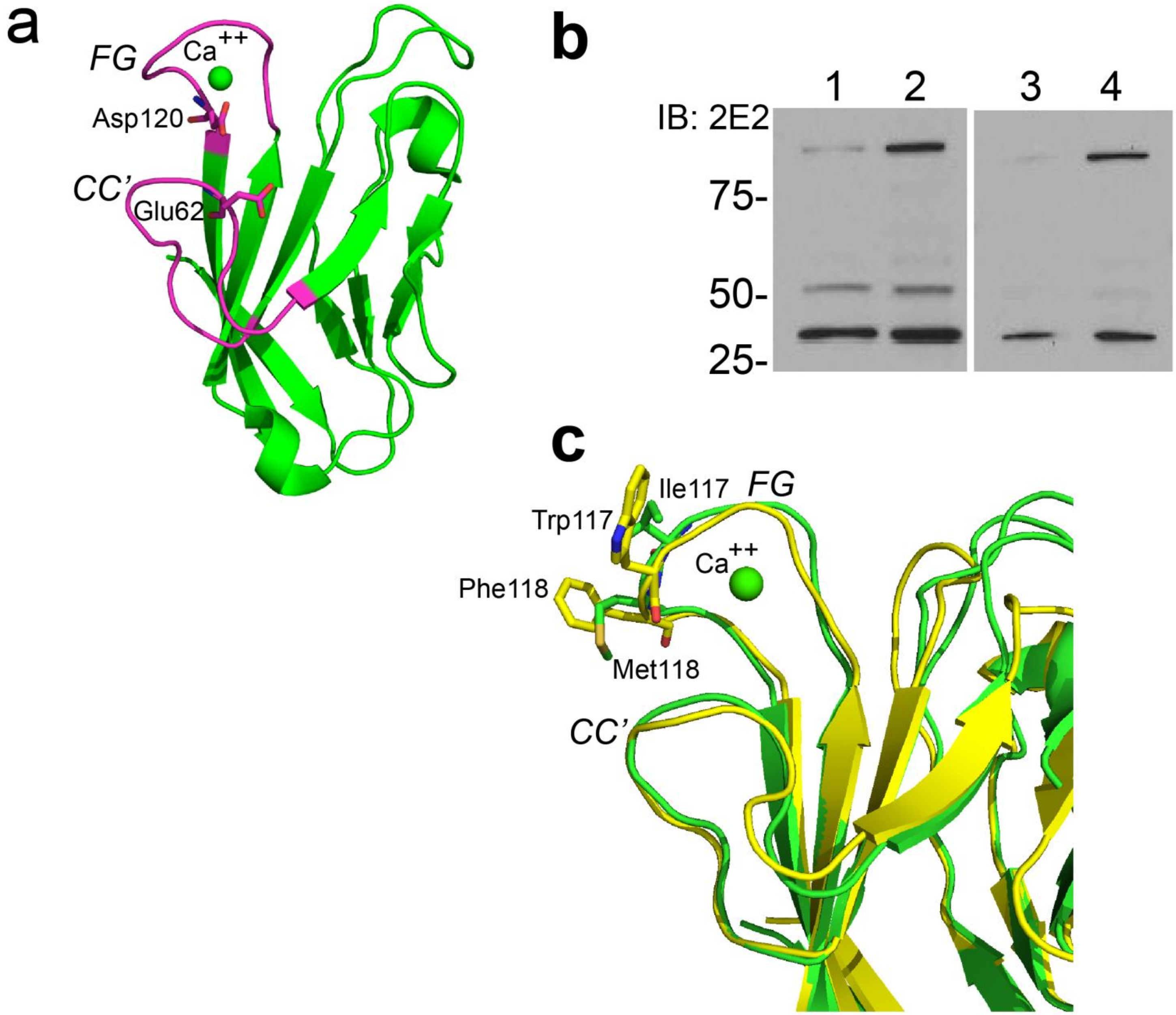

Supplementary Figure 7

Scrambled control peptide

hTIM-3 C-C' loop-derived peptide

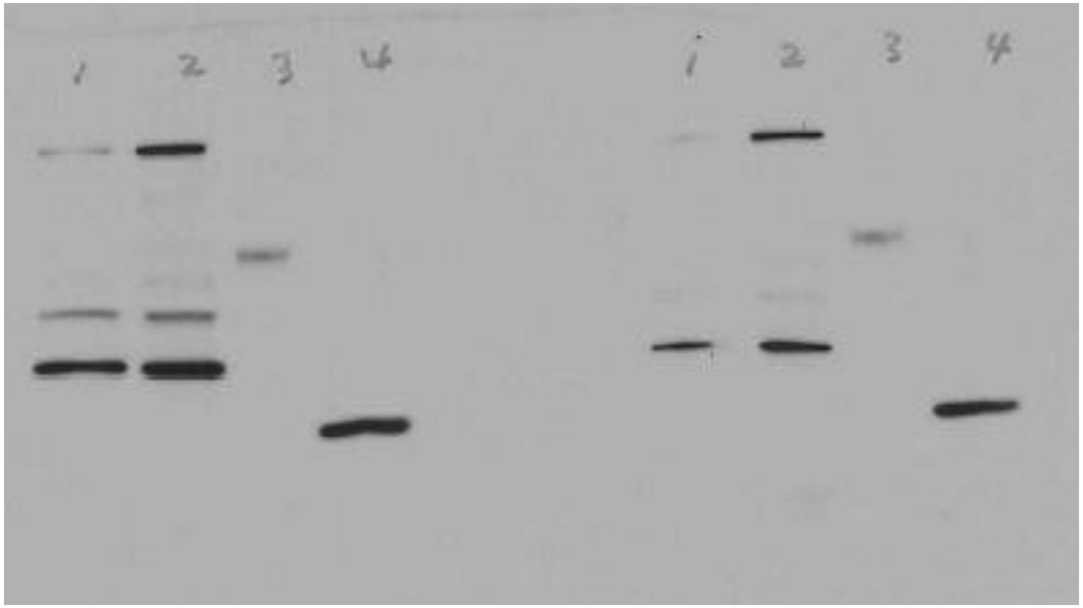

Supplementary Figure 1. Differential scanning fluorimetry of Ca++-bound hTIM-3 IgV. (a) Fluorescence emission with corresponding temperature is plotted for hTIM-3 in the absence (0mM) or presence (5mM, 10mM) of $CaCl_2$. Extrapolated melting temperature (T_m) from the Boltzmann method is depicted in dashed lines for each protein sample. **(b)** Calculated Boltzmann melting temperatures of the fluorescence emission for each corresponding hTIM-3 sample is shown with experimental standard errors from triplicate samples.

Supplementary Figure 2. Structural comparison of hTIM-3, mTIM-3, hTIM-1, and hTIM-4 IgV domains. a, Line diagram of aligned mTIM-3 (Red, PDB code 2OYP) and hTIM-3 IgV (Green, PDB code 6DHB) domains with conserved residues Cys52,Trp53, Cys58, Cys63, Arg81,Val93, Asp104,Cys109, and Cys110 shown in sticks. **b,** B-C loop conformational differences between mTIM-3 (red) and hTIM-3 (green). The conserved Pro37 and Pro50 shown in stick. mTIM-3 residue Pro43 is labeled in italics, and hTIM-3 residues Pro42 and Pro45 are labeled and underlined. **c,** Conformational differences in C” strand and C”-D loop of hTIM-3 (green) and mTIM-3. The hTIM-3 residues Trp78, Thr79, Tyr82, and Trp83 shown in sticks. **d,** B-C and C”-D loop conformation differences among human TIM family, where three additional proline residues (Pro42, Pro45, Pro50) in B-C loop of hTIM-3 (green) are labeled and underlined. Pro37 is labeled and conserved in hTIM-1(yellow), hTIM-3 (green) and hTIM-4 (magenta).

Supplementary Figure 3. Secondary structural comparison of hTIM-3 based on NMR and crystal structure. Top bar diagram shows secondary structure predictions (TALOS+) of hTIM-3 based on $^{15}N/^{13}C$ chemical shift values determined by NMR, defined as fractional probabilities for helix (red), beta-sheet (blue), and loop (gray) respectively. Middle cartoon diagram shows helices colored in red, beta-sheets colored in blue and loops colored in gray based on the TALOS+ secondary structure predictions. Bottom cartoon diagram shows helices colored in pink, beta-sheets colored in yellow and loops colored in gray based on hTIM-3 crystal structure.

Supplementary Figure 4: **Human TIM-3 GFCC' face with bound Ca^{++}.** 2Fo-Fc map is shown at bound Ca^{++} (red sphere) with 1.0 σ (**a**), 3.0 σ (**b**) and 5.0 σ (**c**) levels. Map is superimposed on the final refined model.

Supplementary Figure 5. Structural model of human TIM-3 with bound Ca^{++} and modeled PtdSer. hTIM-3 F-G loop residues Asn119 and Asp120 participates in binding with Ca^{++} and PtdSer. Residues Ile117 and Met118 of hTIM-3 do not participate significantly in PtdSer binding with hTIM-3.

Supplementary Figure 6. Structural mapping and selectivity of anti-human TIM-3 2E2 antibody. a, hTIM-3 CC' and FG loops that contribute to binding of anti-human TIM-3 2E2 antibody as previously determined[1] is highlighted in magenta on a representation of the hTIM-3 crystal structure reported here (PDB code 6DHB) which includes residues Glu62 and Asp120. Bound Ca^{++} is shown by a green sphere. **b,** hTIM-3 C-C' loop-derived peptide peptide (aa 58-77) on right, but not scrambled control peptide on left, inhibits binding of mouse anti-human 2E2 antibody to hTIM-3. Lanes 1 and 2 show immunoblot of 2E2 antibody in the presence of scrambled control peptide, where lane 1 sample is exhausted $CD4^+$ T cells and lane 2 sample is doxycycline induced hTIM-3 expression on transfected Jurkat T cells. Lanes 3 and 4 show immunoblot of 2E2 antibody in the presence of hTIM-3 C-C' loop-derived peptide, where lane 3 sample is exhausted $CD4^+$ T cells and lane 4 sample is doxycycline induced hTIM-3 expression on transfected Jurkat T cells. Full-length blots are presented in Supplementary Figure 7. **c,** Structural alignment of hTIM-3 (green, PDB code 6DHBwith human TIM-1 (yellow, PDB code 5DZO) shows that hTIM-3 residues Ile118 and Met119 are replaced by aromatic residues Trp118 and Phe119 in hTIM-1. Human TIM-3 residues and loops are shown in green whereas hTIM-1 loops and residues are shown in yellow.

Supplementary Figure 7. Full-length blots related to Supplementary fig.6b, where scrambled control peptide lanes 1 and 2 represent lanes 1 and 2 of Supplementary fig.6b, and hTIM-3 C-C' loop-derived peptide lanes 1 and 2 represent lanes 3 and 4 of Supplementary fig.6b.

References

1. Sabatos-Peyton, C. A. *et al.* Blockade of Tim-3 binding to phosphatidylserine and CEACAM1 is a shared feature of anti-Tim-3 antibodies that have functional efficacy. *Oncoimmunology* **7:**e1385690 (2018)

Supplementary Table 1: Human TIM-3 IgV domain secondary structure annotations by crystal structure and NMR

Secondary structure	Corresponding amino acids (Annotated by Crystal structure)	Corresponding amino acids (Predicted by NMR)
A strand	24-VEYRA-28	26-YRA-28
B strand	34-AY-35	34-AYL-36
C strand	51-VCWGK-55	53-WGK-55 (partially assigned)
C' strand	67-VLR-69	unassigned
C" strand	75-VN-76	unassigned
D strand	82-YWL-84	unassigned
First Helix	87-DFRK-90	88-FR-89
E strand	95-LTI-97	95-LTI-97
Second Helix	101-TLAD-104	102-LAD-104
F strand	106-GIYCCRI-111	106-GIYCCRIQ-113
G strand	121-EKFNLKLVIK-130	122-KFNLKLVI-129
